# Inhibition of interleukin-1beta-stimulated dedifferentiation of chondrocytes via controlled release of CrmA from hyaluronic acid-chitosan microspheres

**DOI:** 10.1186/s12891-015-0521-6

**Published:** 2015-03-18

**Authors:** Bei-lei Ma, Pang-Hu Zhou, Ting Xie, Lei Shi, Bo Qiu, Qian Wang

**Affiliations:** Department of Laboratory, Qilu Hospital of Shandong University, 250012 Jinan, China; Department of Orthopedics, Renmin Hospital of Wuhan University, 430060 Wuhan, China; Department of Women Health Care, Hubei Women and Children Hospital, 430070 Wuhan, China; Department of Oncology, Renmin Hospital of Wuhan University, 430060 Wuhan, China

**Keywords:** Hyaluronic acid, Chitosan, Chondrocyte, Dedifferentiation, CrmA, Controlled-released

## Abstract

**Background:**

The previous studies indicated that CrmA could ameliorate the interleukin-1β induced osteoarthritis. In this study, we investigated the controlled-released cytokine response modifier A (CrmA) from hyaluronic acid (HA)-chitosan (CS) microspheres to improve interleukin-1β (IL-1β)-stimulated dedifferentiation of chondrocytes.

**Methods:**

A rat model of osteoarthritis (OA) in vitro was established using 10 ng/ml IL-1β as modulating and chondrocytes inducing agent. HA-CS-CrmA microspheres were added to the medium after IL-1β was co-cultured with freshly isolated rat chondrocytes for 48 hours. The chondrocytes viability and glycosaminoglycan (GAG) content were determined. The level of CrmA secreted was detected by Enzyme-Linked Immunosorbent Assay (ELISA). The protein levels of type II collagen, aggrecan, collagen I and IL-1β were detected using western blotting analyses.

**Results:**

The CrmA release kinetics were characterized by an initial burst release, which was reduced to a linear release over ten days. The production of GAG and the expression of type II collagen, aggrecan significantly increased compared with the control group, while the expression of collagen I and IL-1β decreased.

**Conclusions:**

This study demonstrated that HA-CS microspheres containing CrmA could attenuate the degeneration of articular cartilage by maintaining the phenotype of chondrocytes during culture expansion. The suppression of inflammatory cytokines activity within the joint might be one important mechanism of the action of the microspheres in the treatment of OA.

## Background

Osteoarthritis (OA) is the most prevalent disease of articular joints. Pathophysiologic changes occur in OA cartilage due to the excessive expression of cartilage degrading proteinases, the resultant progressive breakdown of collagen fibers, and the degradation of proteoglycan, mainly aggrecan [1]. IL-1β is considered to play an important role in the pathogenesis of OA, mainly because it can induce the resorption of proteoglycan and type II collagen [2,3]. Consequently, the inhibition of the IL-1β pathway presents a promising means of preventing cartilage degradation during OA pathogenesis. One of the major endogenous inhibitors of the IL-1 pathway is CrmA. CrmA can bond with IL-1β converting enzyme (ICE) (caspase-1) as a pseudosubstrate. This serpin can prevent the proteolytic activation of interleukin-1β, then block the cleavage of pro-IL-1β by ICE thereby suppressing an interleukin-1β response to infection and decreasing the secretion of IL-1β [4,5].

Chitosan (CS), a partially deacetylated derivative from chitin composed of D-gucosamine and N-acetylglucosamine, is structurally similar to GAGs. CS is widely used to elaborate different nanocarriers attributed to the capacity of the polymer to interact with the negatively charged cell surfaces [6]. Many studies have shown its applications in drug, DNA delivery and tissue engineering because of its non-toxicity, biocompatibility and biodegradability [7-10].

Hyaluronic acid (HA) is another biocompatible anionic biopolymer used in a wide array of clinical application. HA is an abundant non-sulfated glycosaminoglycan component of synovial fluid and extracellular matrices and plays an important role in its function. It is involved in cell adhesion, morphogenesis, and inflammation regulation [11]. In osteoarthritis, intra-articular injection of HA can improve the viscoelasticity of synovial fluid, augment the flow of joint fluid, normalize endogenous hyaluronate synthesis, inhibit hyaluronate degradation, reduce joint pain, and improve joint function [12-14]. In our previous study, our results have shown that HA could suppress chondrocyte apoptosis in IL-1β-induced osteoarthritis model in a dose-dependent way [15]. Both of these can be considered as attractive materials for new biocompatible and biodegradable polymers.

In this study, we attempted to combine the virtues of CS and HA in the development of CrmA-loaded microspheres, and intended for attenuate the degeneration of articular cartilage. The interaction between these microspheres and chondrocytes will be investigated, and their potential for preventing OA chondrocytes dedifferentiation evaluated.

## Methods

### Materials

Chitosan (molecular weight:150 kDa, deacetylation:98%), Hyaluronic acid (molecular weight:500-730 kDa), sodium tripolyphosphate (STPP), and IL-1β ELISA kit were provided by Sigma-Aldrich. Cytokine response modifier A (CrmA) was purchaseded from PeproTech. Trypsinase, type II collagenase, DMEM/F12 Medium were purchased from Gibco. All the other chemicals used were of the highest available commercially grade.

### Microsphere preparation and characterization

2 g of chitosan was dispersed into the acetic acid (100 mL) under vigorous stirring for 3 h at ambient temperature (below 20°C) to obtain a transparent chitosan solution (2% w/v), and the hyaluronic acid solution (0.1%, w/v) was obtained using the same method. Then, a desired amount of chitosan solution (10 ml) and hyaluronic acid fluid (5 ml) were immediately dispersed with vigorous stirring to obtain a stable mixture of HA-CS solution. A well-mixed suspension containing 100 mL of paraffin oils and 1 g of Span 80 was dispersed in a reactor and and stirred at 1000rmp for 1 h. 6 mL of HA-CS solution prepared was dropped into the suspension with a speed of 1 ml/min. The suspension in the vessel was stirred at the same speed and temperature for an additional 2 h. Next, 10 ml sTPP solution (10% w/v) was added and kept for an additional 1 h at the same stirring speed. After removing the supernatant liquid paraffin, HA-CS microspheres deposited in the bottom were collected. The microspheres were washed with alcohol and acetone several times to completely remove the residual paraffin oil and Span 80. Microspheres of CS-CrmA, HA-CS-CrmA were prepared. Finally, the microspheres were freeze-dried. The sizes and shapes of the microspheres were examined under a scanning electron microscope (SEM).

### In vitro release profiles

Approximately 20 mg of microspheres, containing CrmA, was dispersed into a phosphate-buffered saline (PBS, PH = 7.4) solution containing 10^4^ U/ml of lysozyme. Next, this solution was placed in a shaking water bath at 37°C at 135 rpm for various time periods, up to ten days. Periodically, the microsphere suspension was centrifuged to collect the supernatant for analysis, followed by the resuspension of microspheres in a fresh PBS containing lysozyme. Samples were assayed for CrmA concentration using ELISA kits (PeproTech, USA) according to the manufacturer’s protocols.

### Chondrocytes isolation and culture conditions

Seven-day-old rats were obtained from the Experimental Animal Center of Wuhan University, China, and were fed under standard conditions (temperature:21 ± 1°C; humidity:55–60%) with food and water continuously available. The care and use of animals followed the recommendations and guidelines of the National Institutes of Health and were approved by the Wuhan University Animal Care and Use Committee.

The cartilage tissues were harvested under sterile conditions from the knees of seven-day-old rats. Then, the cartilage tissues were cut into small pieces (<1 mm^3^) and digested with 0.2% trypsin and 0.2% type II collagenase for 30 min and 2 hours respectively. After washing twice with DMEM, isolated chondrocytes were suspended in DMEM/F12 medium supplemented with 10% fetal bovine serum (FBS) and 1% antibiotics at 37°C with 5% CO_2_. Cell viability was determined using cell viability analyzer (viability > 90%). Primary cells were maintained in monolayer culture throughout the study. After the cells reached confluence, the medium was changed to DMEM/F12 with 0.5% FBS and antibiotics for 6 hours. Then IL-1β (10 ng/ml) was added to the culture medium without rinse for a further 48 hours. The blank group was cultured in DMEM/F12 with 10% FBS without IL-1β, while the control group was only treated with IL-1β. Chondrocytes were divided into five groups cultured in DMEM/F12 containing 10%FBS without antibiotics and incubated for a period of 4 h: A. blank group, B. controls, C. chondrocytes cultured with CS microspheres, D. chondrocytes cultured with HA-CS microspheres, E. chondrocytes cultured with CS-CrmA microspheres, F. chondrocytes cultured with HA-CS-CrmA microspheres. There were 5 samples in each group and each experiment was repeated 5 times.

### Determination of cell viability and GAG synthesis

After 72 hours of co-culture, the microspheres solution was discarded, and the chondrocytes were removed to a fresh media. After standardizing cell samples to one million in different groups using a cell counter, 0.5 mg/ml 3-(4,5-Dimethylthiazol-2-yl)-2,5-dippphenyltetrazolium bromide (MTT) was added to chondrocytes incubated at 37°C with 5% CO_2_ for 4 h. The resulting formation was dissolved in dimethylsulphoxide and absorbance was measured at 570 nm with a microplate reader.

The GAG content in cell supernatants was assessed using Blyscan assay kit (Biocolor, UK), and the chondrocytes were normalized to one million cells in each group. Briefly, cell supernatants were digested enzymatically using proteinase K. Following digestion, a desired amount of supernatant was reacted with Blyscan dye for 30 min. GAG-dye precipitate was obtained by centrifugation and the resulting formation was dissolved in 1 ml dissociation reagent and incubated for 10 minutes. Finally, samples were transferred to a 96-well-plate and absorbance at 656 nm was measured on the spectrophotometer. A standard curve was derived from mixed-isomer shark chondroitin sulfate, and the GAG content was calculated.

### Western blot for type II collagen, Aggrecan, type I collagen and IL-1β

Proteins were extracted from harvested chondrocytes. Protein concentrations were determined using a BCA Protein Assay Kit. After adjusting to equal amounts (50 ul protein per lane) of proteins, they were separated by sulfate-polyacrylamide gel electrophoresis (SDS-PAGE) under reducing conditions and then transferred onto polyvinylidene difluoride membranes. Membranes were blocked in phosphate buffered saline (PBS), pH 7.4, containing 5% nonfat dry milk, and incubated with anti-IL-1β, -type II collagen, Aggrecan, and -type I collagen, respectively. Then incubated with horseradish peroxidase-conjugated secondary antibodies (goat anti-rabbit IgG), followed with visualization by the enhanced chemiluminescence kit.

### Statistical analysis

SPSS 20.0 software was applied for data analysis. Data were presented as the means ± S.D. of five separate experiments. Each experimental condition was represented by triplicate wells, with replicates from each culture averaged and used as one value for analysis. Significant differences among the mean values of multiple groups were evaluated by one-way analysis of variance and Student-Newman-Keuls q-test. P < 0.05 was considered as being significant.

## Results

### Characterization of microspheres

**Figure 1:**
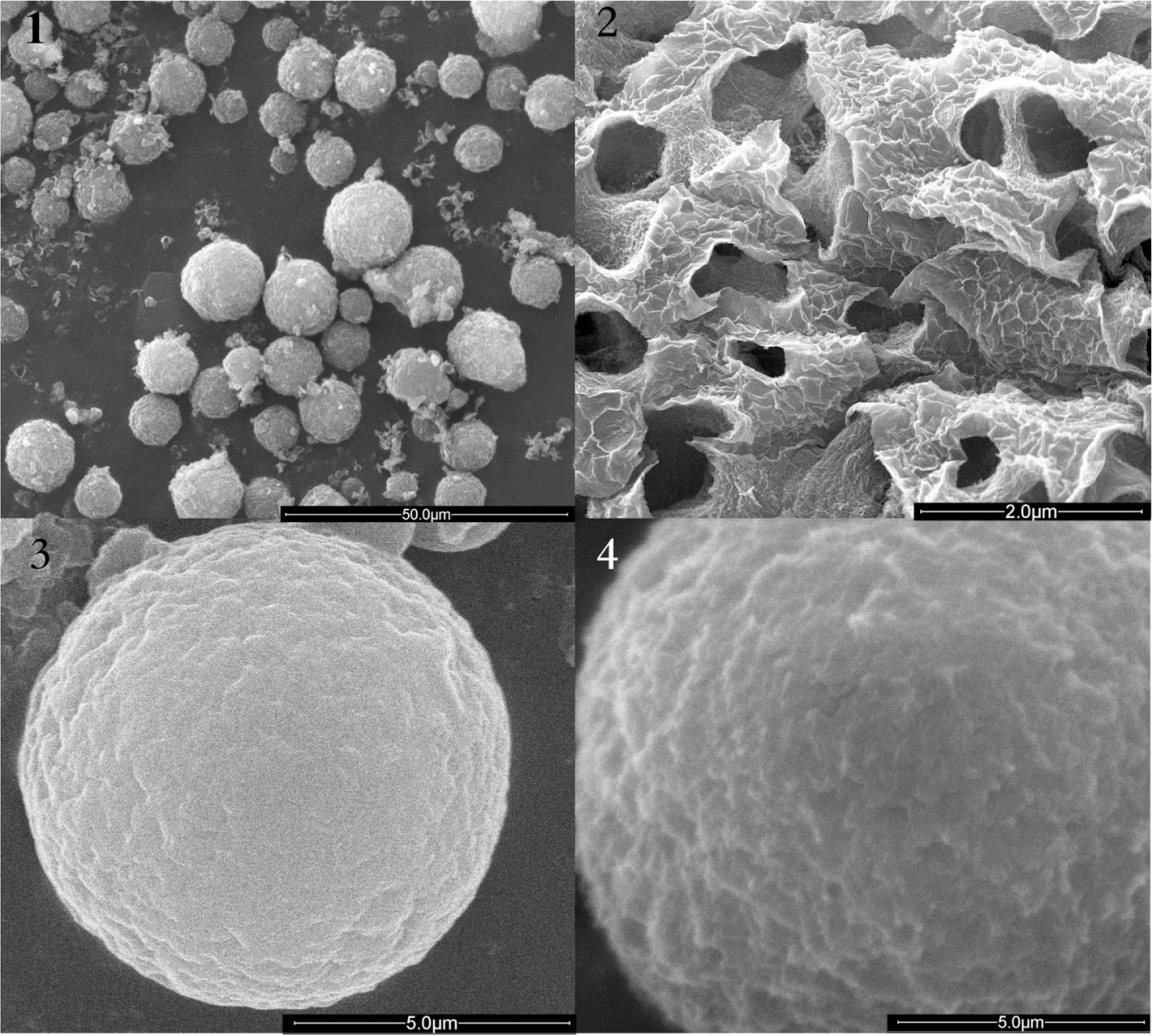
**Characterization of microspheres.** The image showed the characterization of microspheres detected by SEM. 1. Various size of microspheres distributed uniformly. 2. The image of the surfaces and internal of these microspheres. 3.4 There is a slight increase in microsphere size in the presence of HA (Figure 1.3, Figure 1.4).

The HA-CS-CrmA microspheres, fabricated using the emulsification method in the presence of sTPP, were spherical in shape, and the surfaces were smooth. Figure 1 showed the morphology of the microspheres. SEM showed the microspheres were spherical and all the resulting microspheres ranged in size from 8 μm to 15 μm (Figure 1.1). The surfaces of these microspheres appeared to be loose and porous, and the internal structure seemed cellular (Figure 1.2). Regarding the variation in composition and structure, it could be seen that both formulations in the presence and absence of HA presented a gradual increment in the size and morphology. There was a slight increase in microsphere size in the presence of HA (Figure 1.3, Figure 1.4). It was evident that the surface of the microspheres with HA was more porous.

### In vitro release profiles

**Figure 2:**
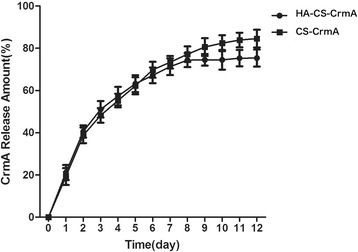
**The release kinetics of HA-CS-CrmA and CS-CrmA microspheres.** In the HA-CS-CrmA group, the release rate of CrmA protein was released lower than CS-CrmA group with the final release of approximately 84% of CrmA. The CrmA from CS-CrmA microspheres was released in a faster continuous manner, with a final release of approximately 75% in the same period. Statistical results represented the mean ± S.D. of five separate experiments. #P < 0.05 VS. blank group;*P < 0.05 VS. Control group.

The release kinetics of CrmA from different microspheres was quite different, depicted in Figure 2. In the HA-CS-CrmA group, the release rate of CrmA protein was released lower than CS-CrmA group. The release kinetics was monitored to 12 days, with the final release of approximately 75% of CrmA within 10 d. Otherwise, the CrmA from CS-CrmA microspheres was released in a faster continuous manner, with a final release of approximately 84% in the same period. This indicated that release kinetics of proteins from the microspheres was influenced by structure of the microspheres. HA, carrying high negative charges which allows the electrostatic interaction with protonated chitosan in an aqueous acidic solution, may result in slow drug release.

### Assay of cell survival rate

**Figure 3:**
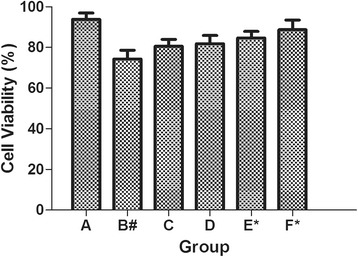
**Viability of chondrocytes in different groups.** The HA-CS-CrmA group presents a highest viability of 88% compared to other control groups. The HA microspheres group (80.5%) produces no statistically significant difference than the CS microspheres group (81%). The statistical results represented the mean ± S.D. of five separate experiments. #P < 0.05 VS. blank group; *P < 0.05 VS. Control group.

The viability of chondrocytes cultured treatment groups was evaluated by measuring their metabolic activity via the MTT assay. Figure 3 displayed the percentage of cell’s viability upon their different treatment. There were three features that were worth mentioning. First, the HA-CS-CrmA group presented a highest viability of 88% compared to other control groups. Second, the fact that HA microspheres group (80.5%) produced no statistically significant difference than the CS microspheres group (81%). Further more, the HA-CS-CrmA group presented a higher viability compared to the CS-CrmA group yielding a percentage of 84.5%, which indicates that CrmA played a crucial role in interaction with chondrocytes.

### GAG contents in cell supernatants

**Figure 4:**
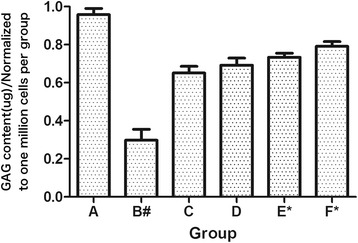
**Total GAG accumulated produced by chondrocytes culture treatment group.** All the treated groups demonstrated an increase in GAG production compared with the IL-1β treated group. And the HA-CS-CrmA group demonstrated significantly greater GAG accumulation as compared to the control group. The statistical results represented the mean ± S.D. of five separate experiments. All data have been normalized to the blank group. #P < 0.05 VS. Blank group; *P < 0.05 VS. Control group

Total GAG quantity was tested to determine which sample group accumulated GAG with greater efficiency, and it was shown in Figure 4. Results showed that all the treated groups demonstrated an increase in GAG production compared with the IL-1β treated group. And, the HA-CS-CrmA group demonstrated significantly greater GAG accumulation as compared to the control group (p < 0.05). However, no statistically significant difference in GAG content was observed between the HA microspheres treated group and the CS microspheres treated group (p > 0.05).

### Western blot for type II collagen, Aggrecan, collagen I and IL-1β

**Figure 5:**
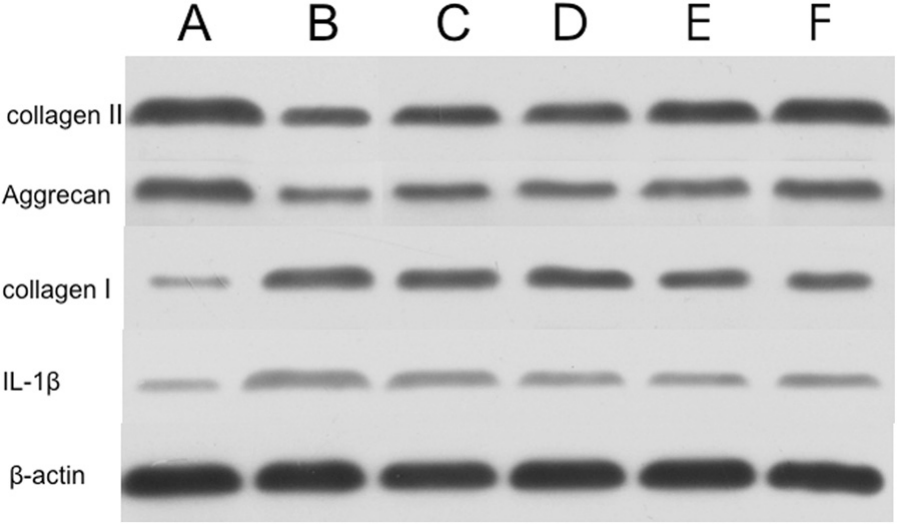
**Western blot analyses for type II collagen, aggrecan I, collagen and IL-1beta.** Type II collagen and aggrecan expression was substantially restored by HA-CS-CrmA microspheres. However, collagen I and IL-1β were decreased in HA-CS-CrmA group and CS- CrmA group compared with the OA group.

As can be seen in Figure 5, type II collagen and aggrecan expression by chondrocyte, which had been significantly reduced by IL-1β exposure, was substantially restored by HA-CS-CrmA microspheres. However, collagen I and IL-1β decreased in HA-CS-CrmA group and CS- CrmA group compared with the OA group. The results indicated that the chondrocyte changed to normalization when treated with microspheres, especially in the presence of CrmA.

## Discussion

This study investigated that the feasibility of the novel microspheres containing CrmA as a drug release system to effectively interact with chondrocytes, produce therapeutic levels of ligand, ameliorate the dedifferentiation of chondrocytes. Our results demonstrated that cell viability, and ECM production such as GAGs and phenotype of the chondrocytes cultured with microspheres evolved normalization compared with OA group. Based on previous reports and our results, we reasonably deduce that the control-released CrmA from hyaluronic acid-chitosan microspheres has a great potential as a new way to protect chondrocyte in the process of OA.

In our study, one of the main factors affecting our results was the microsphere structure. Comparing the CS-CrmA group with HA-CS-CrmA group, it was notable that HA conjugation could enhance the ability of the microspheres to interact with the chondrocytes and to controlled-released drug. Collagen II, GAG and aggrecan seemed more prominent in HA-CS-CrmA microspheres. Moreover, collagen I and IL-1β downregulation had previously been shown in chondrocytes treated with HA-CS-CrmA microspheres. It has been reported that HA treatment may prevent the IL-1β-induced downregulation of collagen II and proteoglycan in OA chondrocytes by blocking collagenases, so as to decelerate the progression of OA [16]. Meanwhile, the control-released action was demonstrated to last much longer in the presence of HA. More importantly, the efficiency of this release manner was improved by 12%. This may have been due to the high negative charges and the very highly swollen gel-like characteristic of HA [17]. HA may promote controlled-release by loosening the CS-protein binding [18]. In addition, HA is an adhesion modulator molecule, which can mediate the early stage of cell-substrate interaction [19]. Furthermore, CD44 is known as a cell surface receptor for HA internalization and turnover [20,21]. CD44 is a transmembrane glycoprotein expressed in a variety of cell types in connective tissues and a major cell surface protein in chondrocytes, and is highly expressed in inflammatory conditions [22]. This has been postulated to have a function as the principal receptor for HA. Anti-CD44 treatment using IM7 antibody has been reported to activate some intracellular signaling pathways that block the action of IL-1β [23]. Therefore, the application of HA as a component of the microspheres might be a reasonable approach for enhancing the interaction with chondrocytes in OA. Simultaneously, the presence of HA could allow HA combining with CS through electrostatic interaction [24]. It was observed that the size of the microspheres was strongly influenced when HA was included in the microspheres.

GAG is a kind of polysaccharide, which together with collagen type II forms the main components of the cartilage matrix. Secretion of GAGs by in vitro cultured chondrocytes can be regarded as a sign that the chondrocytes phenotype is maintained [25]. TypeIIcollagen and aggrecan could maintain the chondrocyte phenotype. This study demonstrated that microspheres were able to prevent the IL-1β–induced breakdown of type II collagen and proteoglycan in OA cartilage by decreasing the secretion of IL-1β, leading to the deceleration of OA progression. According to figures of Figure 3, 5 and Table 1, we could deduce that cell viability correlated very well with these chondrogenic markers. As shown in Table 1, the expression of IL-1β and collagen I decreased, while GAG, collagen type II, aggrecan production significantly increased in HA-CS-CrmA group and CS-CrmA group, but not signally in HA-CS group and CS group. These data indicated that CrmA played a key role in the microspheres in preventing the chondrocyte dedifferentiation. CrmA is the natural inhibitor of ICE (caspase-1), whose amino-acid sequence is similar to Acetyl-Asp-Glu-Val-Asp-aldehyde (Ac-YVAD-CHO), a caspase-1-specific inhibitor [26]. Caspase-1 has great specificity for cleaving pro-IL-1β, thus decreasing the secretion of IL-1β [27,28]. The suppression of inflammatory cytokines activity within the joint might be one important mechanism of the clinical action of the microspheres in the treatment of OA. Since CrmA has not been studied widely in the field of OA, it shows the advantages in the application of protection of the chondrocyte in OA.

**Table 1:** **The protein expressions of collagenII, Aggrecan, collagenI and IL-1β**

**Group**	**CollagenII**	**Aggrecan**	**CollagenI**	**IL-1β**
A	1.14 ± 0.07	1.26 ± 0.12	0.12 ± 0.03	0.30 ± 0.04
B	0.55 ± 0.12^#^	0.50 ± 0.06^#^	0.51 ± 0.10^#^	0.67 ± 0.02^#^
C	0.76 ± 0.03	0.75 ± 0.03	0.48 ± 0.04	0.59 ± 0.10
D	0.78 ± 0.08	0.76 ± 0.13	0.44 ± 0.06	0.57 ± 0.07
E	0.92 ± 0.05*	0.89 ± 0.08*	0.36 ± 0.08*	0.49 ± 0.11*
F	0.97 ± 0.04*	1.02 ± 0.05*	0.32 ± 0.11*	0.34 ± 0.06*

The statistical results represented the mean ± S.D. of five separate experiment. Type II collagen and aggrecan expression was substantially restored in HA-CS-CrmA group. However, collagen I and IL-1β were reduced in HA-CS-CrmA group and CS- CrmA group compared with the OA group.

#P < 0.05 VS. blank group; *P < 0.05 VS. control group.

For the successful interaction with OA chondrocytes, we prepared the HA-CS microspheres containing CrmA. It was revealed that the sustained release of CrmA from HA-CS microspheres could attenuate the degeneration of articular cartilage by maintaining the phenotype of chondrocyte during culture expansion. The data derived from this study suggest great promise to utilize HA-CS microsphere as CrmA carriers for protection of the chondrocyte in the process of OA.

## Conclusions

In conclusion, our previous study has demonstrated that HA could suppress chondrocyte apoptosis in IL-1β-induced osteoarthritis model in a dose-dependent way. In this study, for the first time, we found that the release kinetics of CrmA were characterized by an initial burst release, and the production of GAG and the expression of type II collagen, aggrecan significantly increased compared with the control group, while the expression of collagen I and IL-1β decreased. These results indicated that CrmA could attenuate the degeneration of articular cartilage by maintaining the phenotype of chondrocytes during culture expansion. The suppression of inflammatory cytokines activity within the joint might be one important mechanism of the clinical action of the microspheres in the treatment of OA.
